# Lower extremity isokinetic strength characteristics of amateur boxers

**DOI:** 10.3389/fphys.2022.898126

**Published:** 2022-08-10

**Authors:** Zixiang Zhou, Chao Chen, Xin Chen, Wenjuan Yi, Weijia Cui, Rui Wu, Dexin Wang

**Affiliations:** ^1^ School of Physical Education and Sport Training, Shanghai University of Sport, Shanghai, China; ^2^ Shanghai Key Lab of Human Performance, Shanghai University of Sport, Shanghai, China; ^3^ Department of Sport and Health Sciences, Technical University of Munich, Munich, Germany

**Keywords:** knee strength, isokinetic, bilateral ratios, unilateral ratios, functional ratios

## Abstract

**Aim:** Sufficient strength and dynamic stability of the lower limbs are essential for improving punching force and preventing injury in amateur boxers. However, there are still no comprehensive reports on the isokinetic knee strength of boxers with different performance levels. The current study aimed to profile the isokinetic muscle strength of flexion and extension of the knee in boxers, as well as bilateral, unilateral, and functional ratios, and investigate the variation in these muscle strength characteristics associated with different performance levels.

**Methods:** Boxers were divided in two performance groups, elite (five males and four females) and non-elite groups (five males and four females). Muscle strength of the knee was determined *via* an IsoMed2000 device. Parameters examined included peak torque of the hamstring (H) and quadriceps (Q) during concentric (con) and eccentric (ecc) contractions at low (60°/S), medium (180°/S), and high (240°/S) speed and bilateral ratios (BLs), unilateral ratios (ULs), and functional ratios of dominant (D) and non-dominant limbs.

**Results:** In all angular velocities, the peak torque of H and Q was stronger in the elite group than in the non-elite group. ULs were lower in the elite group than in the non-elite group in Hcon/Qcon at 180D (*p* < 0.01) and 180ND (*p* < 0.05) and in Hecc/Qecc at 60D, 180D (*p* < 0.05) and 240D (*p* < 0.01). The elite group had higher BLs than the non-elite group in Hcon at 60°/S (*p* < 0.05) and Qcon at 180°/S (*p* < 0.05). The non-elite group had a higher functional ratio than elite boxers in Hecc/Qcon at 180D and 240D (*p* < 0.01).

**Conclusion:** Elite boxers had stronger knee strength in con and ecc contractions. All boxers had normal Hecc/Qecc and Hcon/Qecc. Hcon/Qcon and Hecc/Qcon were abnormal at lower angular velocity. Elite boxers had higher BLs and lower ULs, indicating that they are at a higher risk of injury.

## Introduction

Amateur boxing was included in the Olympic program in 1904. In high-level amateur boxing, well-developed muscle strength of the upper and lower limbs is necessary (Chaabène et al., 2015). The strength of the lower extremities plays a central role in punching performance, by transferring the linear momentum of force from the lower limbs to the trunk and subsequently to the upper limb (Loturco et al., 2014). Maximum leg strength is reportedly correlated with the Jab (0.68) and with the Cross (0.83) (Loturco et al., 2016). Boxers require the capacity to sustain an activity rate of 1.4 actions per second in the competition, consisting of 20 punches, 2.5 defensive movements, and 47 vertical hip movements per minute (Davis et al., 2013). The hamstring (H) and quadriceps (Q) are crucial primary movers during these motions. Lower limb strength deficits affect performance, and a high correlation between lower extremity strength and punch performance has been reported (Loturco et al., 2016). More importantly, at a certain exercise intensity, the athlete exceeds the mechanical limitations of the muscle unit, and muscle strength deficits and asymmetries lead to hamstring injury (Askling et al., 2003; Dauty et al., 2003). The major functions of Q are acceleration and control of flexion activity. The primary roles of H are knee stabilization during acceleration and changing the direction of motion (Stølen et al., 2005). The anterior cruciate ligament (ACL) stabilizes the knee by limiting anterior translation of the tibia to the femur with the help of hamstrings (Kannus, 1988b; Pettitt et al., 2002). Excessive anterior translation can occur during dynamic exercise if the quadriceps are stronger than the hamstrings and the ACL is subjected to greater shear stresses. When the hamstring fails to provide adequate strength to slow knee rotation or reduce anterior tibial shear stresses, (Coombs and Garbutt, 2002), both the hamstring and the ACL become more susceptible to injury (Petersen and Hölmich, 2005; Holcomb et al., 2007).

The overall incidence of lower extremity injury is 1,220 per 1,000 h in competition. Boxers may suffer injury due to overuse of the lower extremities, and the total rate of injury is 21.6%. Although the injury severity scores are not high, strains and sprains of the knee are common in boxers (Jordan and Campbell, 1988; Zazryn et al., 2006). A meta-analysis noted that overtraining is a severe issue for combat athletes, and the risk of muscle injury increases with increasing training duration and intensity (Lystad et al., 2014). During training and competition, boxers have to repeatedly change direction, maintain balance, and move rapidly *via* concentric (con) and eccentric (ecc) muscle contractions. Muscle strength deficit and incorrect agonist–antagonist muscle ratios have been identified as major variables, predisposing athletes to lower limb injury (Namazi et al., 2019). Strength asymmetry (i.e., Hcon/Qcon ratios <0.6) of the hamstrings and quadriceps may increase the likelihood of ACL injury (Yeung et al., 2009; Orchard et al., 2012). In addition to ACL injury, muscle strength imbalance also causes the patella to move laterally, resulting in pain and injury on the lateral side of the patellofemoral joint (Cowan et al., 2009). Higher bilateral asymmetry values (bilateral strength difference >15%) in the dominant limb (D) and non-dominant limb (ND) can be related to a higher possibility of injury in weak limbs (Croisier et al., 2002; Zvijac et al., 2014). Stastny et al. reported that imbalances in unilateral (Hcon/Qcon and Hecc/Qecc) or bilateral (D and ND muscles) strength increased the risk of soft tissue injury of the knee (Stastny et al., 2014). It is known that co-activation of the hamstring and quadriceps occurs with opposing contraction modes. The role of the hamstring is to decelerate the lower limb during rapid and powerful con contractions of the quadriceps with protective ecc contraction (Coombs and Garbutt, 2002). Accordingly, Coombs et al. suggest evaluating ecc strength and describing imbalances using functional ratios, including the Hecc/Qcon ratio and the Hcon/Qecc ratio. In one report, it was concluded that an Hecc/Qcon ratio less than 1.0 may increase the risk of knee injuries (Coombs and Garbutt, 2002).

Keeping athletes healthy is critical for improving their performance. It has been reported that repeating the same movement patterns for many years can have negative effects, which usually present as contralateral and ipsilateral muscle strength imbalances (Tourny-Chollet et al., 2009; Lisowska et al., 2020). Elite boxers have a higher activity profile, performing more offensive and defensive actions (Davis et al., 2013). Longer training times and the more frequent activity rate exhibited by elite boxers may increase their risk of injury. Systematic assessment of isokinetic muscle strength is essential with respect to both performance and health in order to optimize training programs and prevent injury (Croisier et al., 2008). However, until now, only one study has investigated the isokinetic shoulder characteristics of amateur boxers (Tasiopoulos et al., 2018). A comprehensive evaluation of the isokinetic characteristics of the lower limb in boxers is lacking. The current study aimed to assess isokinetic knee strength in boxers with different performance levels, including the peak torque of hamstrings and quadriceps in con and ecc modes, side-to-side asymmetry, and H/Q conventional ratios and functional ratios. We hypothesized that peak hamstring and quadricep torque in con and ecc would be stronger in an elite group than in a non-elite group at all angular velocities.

## Materials and methods

Boxers were divided in two performance groups depending on the level of their competition division: Elite (males = 5, females = 4, national-level competition), non-elite group (males = 5, females = 4, provincial-level competition) (Table 1). Based on the study by Ahmadi et al. (2020), sample size calculation was conducted by *a priori* analysis using G*power software (3.1; University of Dusseldorf, Dusseldorf, Germany) with the methods of repeated-measures ANOVA between factors (number of groups: 2, number of measurements: 3), with statistical power = 0.91, α level = 0.05, and effect size = 0.8. A total sample size of 14 subjects (seven per group) would be required. The sample size used in this study was sufficient to detect a prespecified effect size. Rear hand punch and rear leg were identified as dominant limbs. All of the players self-reported the right limb as their dominant limb. Participants reported no history of knee injury within 6 months prior to testing. They were informed about the procedure and the aim of the study, and subsequently they provided their written consent for participation. Ethical consent was provided by the Shanghai University of Sport Research Ethics Committee (approval number:102772021RT029) and in accordance with the Helsinki declaration.

**TABLE 1:** Physical characteristics and training experience of boxers by the performance group.

Group	Age (yrs)	Height (cm)	Weight (kg)	Training experience
Elite	23.3 ± 0.7	170.2 ± 4.7	65.3 ± 5.7	6.6 ± 0.5
Non-elite	21.1 ± 0.9	169.7 ± 5.3	64.8 ± 6.0	4.7 ± 0.5

**FIGURE 1:**
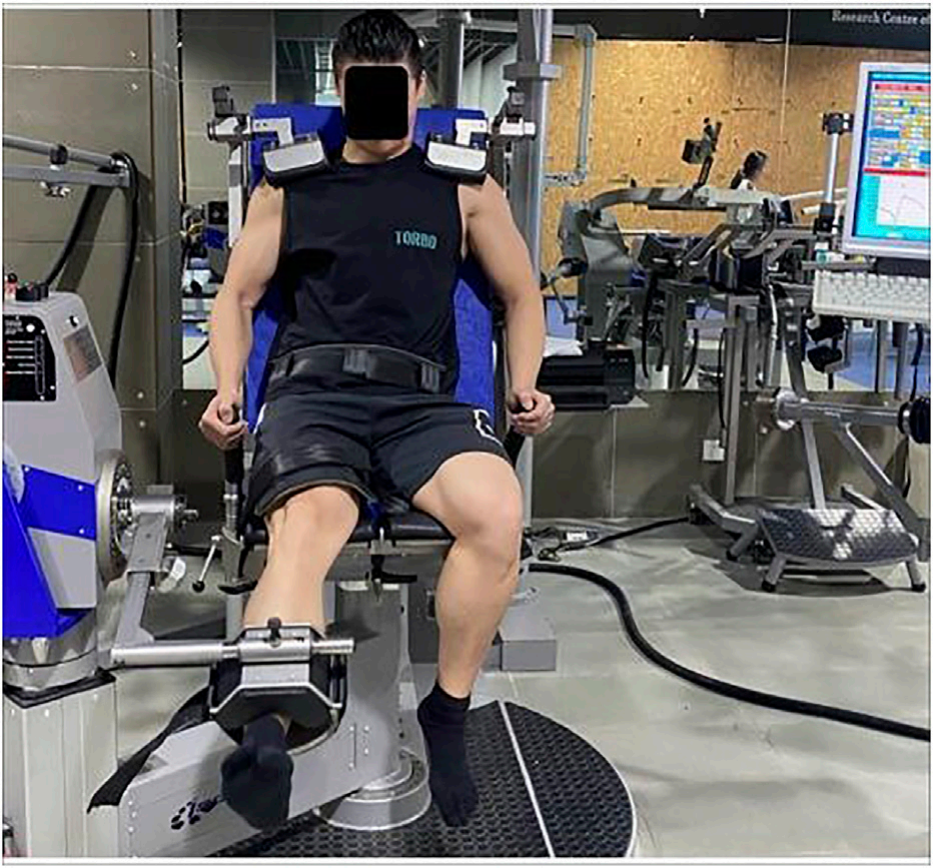
Isokinetic knee testing.

The maximum torque of the hamstrings and quadriceps was measured by an isokinetic dynamometer IsoMed 2000 (Germany) at 60°/s,180°/s and 240°/S (Figure 1), which showed high reliability of the knee test (ICC = 0.90–0.96) in a prior study (Chen et al., 2021). Before each test, athletes performed a 15–20 min warm-up including jogging and stretching quadriceps, hamstrings, adductors, iliopsoas, and gluteus. According to the instruction manual for the IsoMed2000 Systems, the participant was seated in appropriate and stable position using fixed straps to avoid compensatory trunk movement. The rotation axis of the machine and the knee joint were aligned, and gravity compensation was performed (Hamilton et al., 2014). Prior to testing, subjects performed five contractions in order to familiarize with the session (Chan et al., 2020) and check for straps firmly fixed to the trunk, waist, and thigh to minimize secondary joint movement. After a 5-min recovery period, during continuous (bidirectional) knee flexion–extension movements, the isokinetic torque of the quadriceps and hamstrings was recorded at the angular velocities of 60°/s, 180°/S, and 240°/s (slow to fast). The range of motion (ROM) for testing was set from 10° to 90° of flexion (0° = full extension) (Brown et al., 2014; Xaverova et al., 2015). Every con maximum contraction was measured the first day, followed by the measurement of an ecc the next day. Five maximal flexions and extensions were performed at the three different angular velocities with 1-minute recovery (Andrade et al., 2013; Ruas et al., 2015). Five-minute rest was given between the measurement of both the knees. During the test, the participants must develop the maximum force at the same time during the two phases of extension and flexion. The test began with the D limb and then the ND limb. The test was conducted at approximately 10 (AM) over a period of 1 month from September–October 2021.

Outcome measures were relative peak torque (RPT) of con and ecc contractions of D and ND limbs and bilateral, unilateral, and functional ratios. BLs was calculated using formula 100 × [PT(D) - PT (ND)]/PT(D)](Maly et al., 2015). The ULs was calculated by dividing hamstring con (ecc) peak torque by quadricep con (ecc) peak torque. Functional ratios were considered Hecc/Qcon and Qecc/Hcon.

### Statistical analysis

Descriptive statistics (means and standard deviations) were performed to summarize all data. Averages were used for further analysis. Data were checked for normal distribution by the Shapiro–Wilk test. Two-way (2 × 3) repeated measures ANOVA was used to analyzed the date (group: elite and non-elite, Angular velocities: 60°/S, 180°/S, and 240°/S). Bonferroni posthoc analysis was used for pairwise comparisons. Independent *t*-tests were performed to analyze the BLs differences between performance groups. The effect size was reported using Cohen’s *d*. An effect size<0.2 was considered minimal, 0.2–0.5 small, 0.5–0.8 moderate, and >0.8 large (Cohen, 1988). The significance level was set at a *p*-value <0.05. All statistical analyses were performed using SPSS 26.0 software (IBM Corp. Amork, NY) and PRISM (GraphPad Software, Inc. Version 9.0.2 for Windows).

## Results

### Peak torque

**FIGURE 2:**
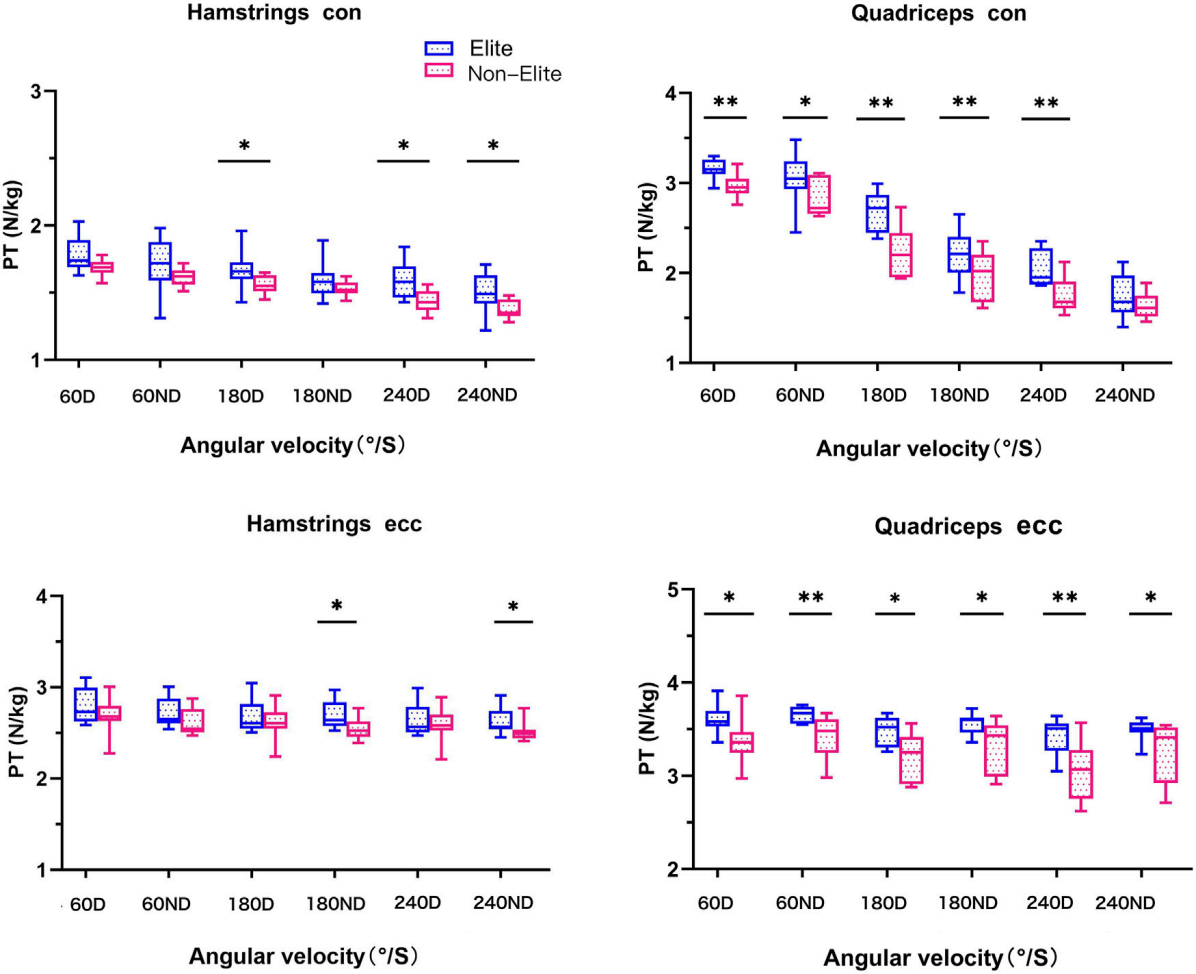
Relative peak torque (RPT) of hamstring (H) and quadriceps (Q) of the knee against 60°/S, 180°/S, and 240°/S in boxers. Con, concentric; ecc, eccentric; D, dominant limb; ND, non-dominant limb; differences between the elite and non-elite group were presented as *p < 0.05, **p < 0.01.

The relative peak torque (RPT) of all boxers can be seen in Tables 2, 3 and Figure 2. A large main effect of performance group on PT was found (*p* < 0.01) with the elite group showing larger values in Qcon at 60D (*d* = 1.58), 180D (*d* = 1.78), 240D (*d* = 1.44), and 180ND (*d* = 0.89) and Qecc at 60ND (*d* = 1.31) and 240D (*d* = 1.43).

**TABLE 2:** Quadriceps and hamstring strength on both sides of the limb for the elite group.

Muscle	Variable	60°/S	180°/S	240°/S
Quadriceps	Extensor con D	3.15 ± 0.11	2.68 ± 0.21	2.03 ± 0.20
	Extensor ecc	3.61 ± 0.15	3.48 ± 0.16	3.41 ± 0.19
	Extensor con ND	3.04 ± 0.30	2.21 ± 0.28	1.76 ± 0.24
	Extensor ecc	3.65 ± 0.12	3.55 ± 0.11	3.49 ± 0.11
Hamstrings	Flexor con D	1.78 ± 0.13	1.67 ± 0.14	1.59 ± 0.14
	Flexor ecc	2.80 ± 0.21	2.68 ± 0.18	2.64 ± 0.18
	Flexor con ND	1.71 ± 0.20	1.59 ± 0.14	1.50 ± 0.15
	Flexor ecc	2.72 ± 0.17	2.69 ± 0.16	2.63 ± 0.14

Relative Values are mean ± standard deviation; D: dominant limb, ND: non-dominant limb.

**TABLE 3:** Quadriceps and hamstring strength on both sides of the limb for the non-elite group.

Muscle	Variable	60°/S	180°/S	240°/S
Quadriceps	Extensor con D	2.96 ± 0.13	2.24 ± 0.28	1.75 ± 0.19
	Extensor ecc	3.37 ± 0.24	3.21 ± 0.25	3.05 ± 0.30
	Extensor con ND	2.82 ± 0.21	1.96 ± 0.28	1.63 ± 0.14
	Extensor ecc	3.41 ± 0.23	3.32 ± 0.27	3.24 ± 0.32
Hamstrings	Flexor con D	1.69 ± 0.09	1.56 ± 0.08	1.43 ± 0.11
	Flexor ecc	2.69 ± 0.20	2.62 ± 0.17	2.59 ± 0.18
	Flexor con ND	1.62 ± 0.07	1.53 ± 0.12	1.37 ± 0.09
	Flexor ecc	2.63 ± 0.15	2.55 ± 0.12	2.51 ± 0.11

Relative Values are mean ± standard deviation; D, dominant limb; ND, non-dominant limb.

Furthermore, a large main effect of the performance group on PT was shown (*p* < 0.05), with the elite group showing larger values in Qcon at 60ND (d = 0.85); Hcon at 180D (*d* = 0.96), 240D (d = 1.27), and 240ND (*d* = 1.05); Hecc at 180ND (*d* = 0.99) and 240ND (*d* = 0.95); and Qecc at 60D (*d* = 1.20), 180D (*d* = 1.29), 180ND (*d* = 1.12), and 240ND (*d* = 1.04).

No difference (*p* > 0.05) between the performance groups was observed in Qcon at 240ND (*d* = 0.66), Hcon at 60D (*d* = 0.80), 60ND (*d* = 0.60), and 180ND (*d* = 0.46), and Hecc at 60D (*d* = 0.54), 180D (*d* = 0.34), 240D (*d* = 0.28), and 60ND (*d* = 0.56).

### Bilateral ratios

**FIGURE 3:**
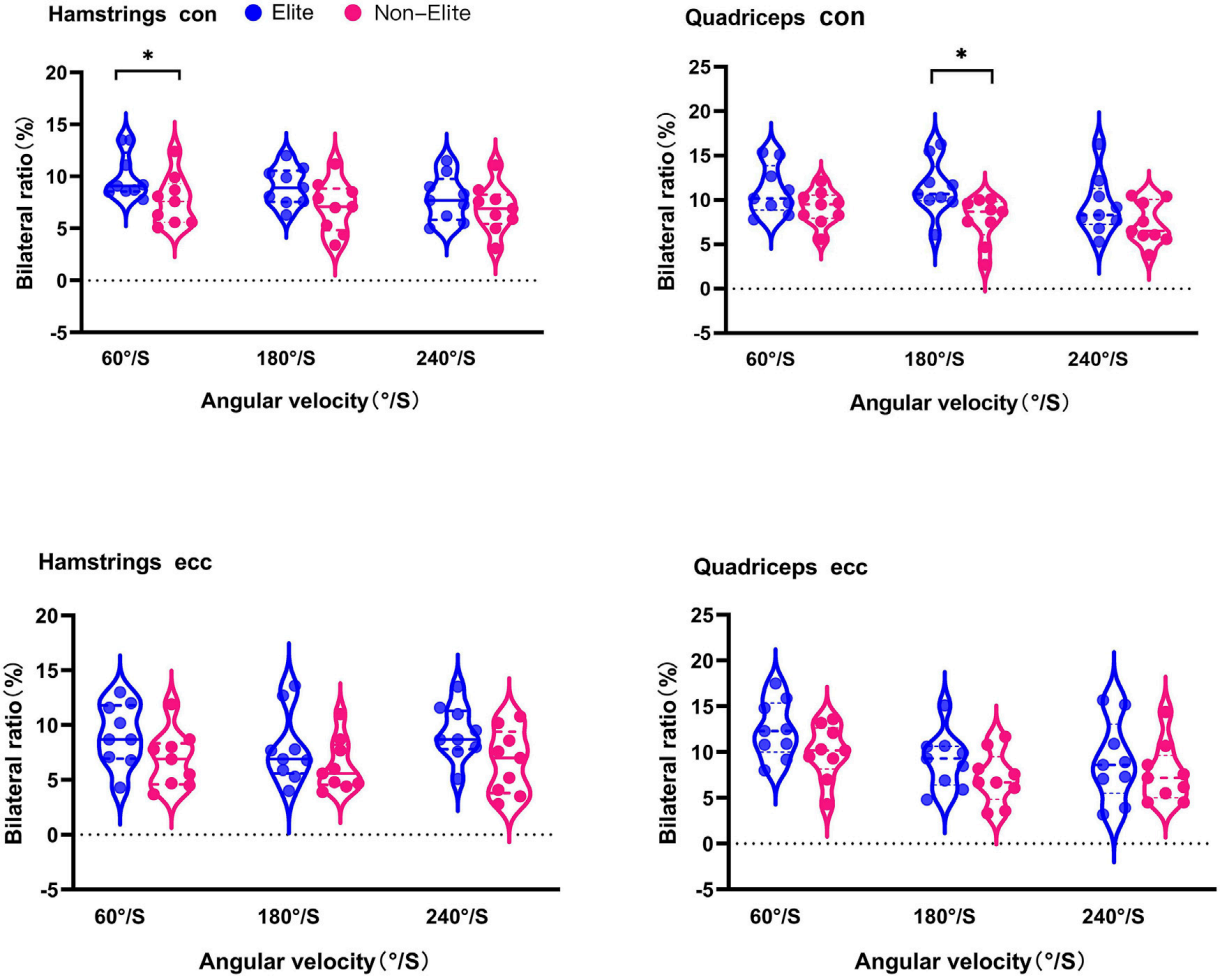
Bilateral ratio of hamstring (H) and quadriceps (Q) of the knee against 60°/S, 180°/S, and 240°/S in boxers. Differences between elite and non-elite groups were presented as *p < 0.05.

The bilateral ratio of all boxers is shown in Table 4; Figure 3. The elite group had a higher BLs than the non-elite group in Hcon at 60°/S (*p* < 0.05, *d* = 1.01) and Qcon at 180°/S (*p* < 0.05, *d* = 1.29). Elite boxers had higher BLs than the non-elite group in the other speeds, whereas no significant difference was found (*p* > 0.05) in Hcon at 180°/S and 240°/S (*d* = 0.89, 0.43), Qcon at 60°/S and 240°/S (*d* = 0.82, 0.70), Hecc at 60°/S, 180°/S, and 240°/S (*d* = 0.85, 0.55, and 0.98, respectively), and Qecc at 60°/S, 180°/S, and 240°/S (*d* = 0.82, 0.64 and 0.34, respectively).

**TABLE 4:** Bilateral ratios (%) in extensor and flexor by the performance group.

Group	Variable	60°/S	180°/S	240°/S
Elite	Quadriceps con	11.08 ± 2.77	11.38 ± 3.07	9.36 ± 3.28
	Quadriceps ecc	12.43 ± 3.13	9.08 ± 3.08	8.98 ± 4.38
	Hamstrings con	10.00 ± 2.18	9.04 ± 1.84	7.89 ± 2.20
	Hamstrings ecc	9.16 ± 2.82	7.86 ± 3.24	9.30 ± 2.47
Non-elite	Quadriceps con	9.13 ± 1.92	7.76 ± 2.53	7.37 ± 2.36
	Quadriceps ecc	9.94 ± 2.96	7.19 ± 2.83	7.69 ± 3.21
	Hamstrings con	7.70 ± 2.39	7.11 ± 2.45	6.93 ± 2.28
	Hamstrings ecc	6.86 ± 2.58	6.31 ± 2.36	6.64 ± 2.92

All data are presented as mean ± standard deviation.

### Unilateral ratios

**FIGURE 4:**
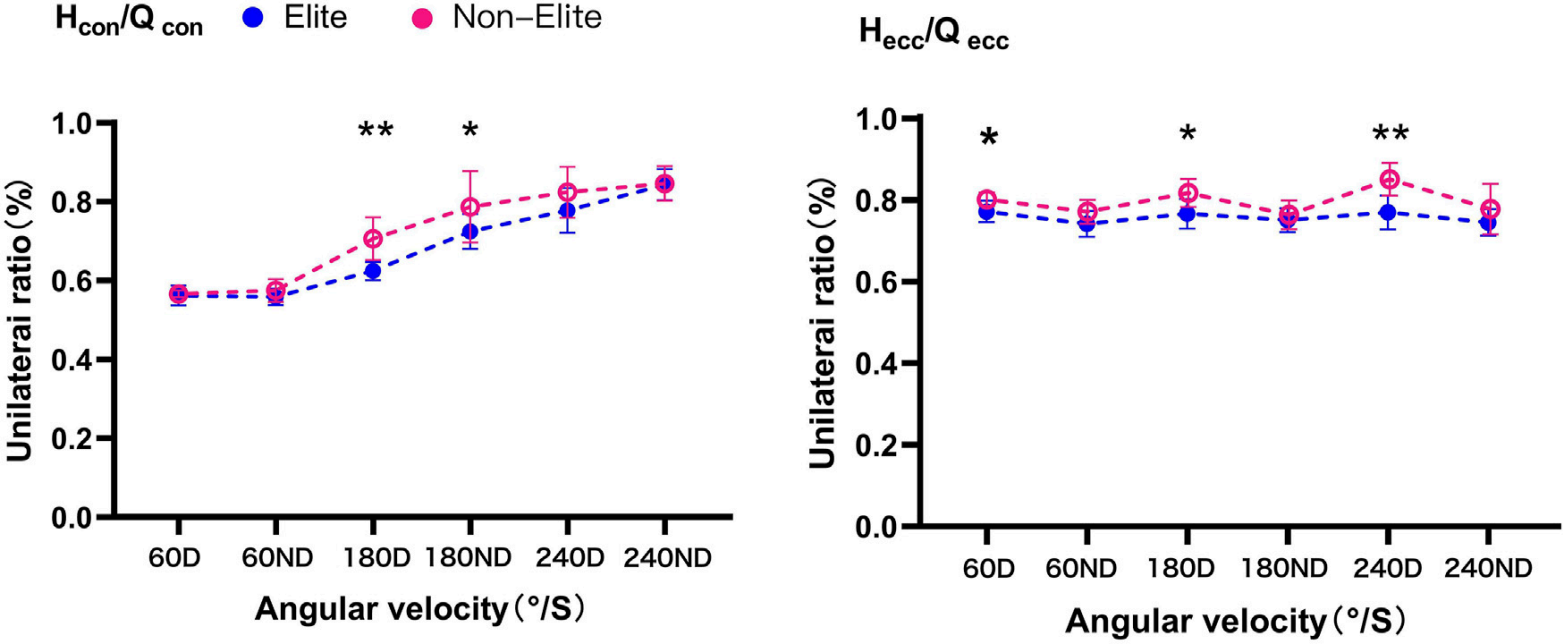
Unilateral ratio of hamstring (H) and quadriceps (Q) of the knee against 60°/S, 180°/S, and 240°/S in boxers. Differences between the elite and non-elite group were presented as *p < 0.05, **p < 0.01; D, dominant limb; ND, non-dominant limb.

The unilateral ratio is shown in Figure 4. Hcon/Qcon was lower in the elite group than in the non-elite group at 180D (*p* < 0.01, *d* = 2.10) and 180ND (*p* < 0.05, *d* = 1.55). No difference (*p* > 0.05) between the performance groups was observed in 60D (*d* = 0.39), 240D (*d* = 0.72), 60ND (*d* = 0.39), and 240ND (*d* = 0.25).

Hecc/Qecc was significantly lower in the elite group than in the non-elite group at 60D (*p* < 0.05, *d* = 1.50), 180D (*p* < 0.05, *d* = 1.33), and 240D (*p* < 0.01, *d* = 2.00). No difference (*p* > 0.05) between the performance groups was found in ND at three velocities (*d* = 1.01, 0.33, and 0.63, respectively).

### Functional ratios

**FIGURE 5:**
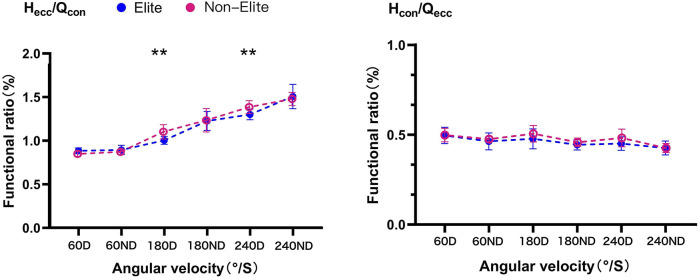
Functional ratio against 60°/S, 180°/S, and 240°/S in boxers. Differences between the elite and non-elite group were presented as *p < 0.05, **p < 0.01; D, dominant limb; ND, non-dominant limb.

The functional ratio is shown in Figure 5. The elite group had lower Hecc/Qcon ratio than the non-elite group at 180D (*p* < 0.01, *d* = 2.84) and 240D (*p* < 0.01, *d* = 2.76). No difference (*p* > 0.05) between the performance groups was observed in 60D (*d* = 1.00) and ND at three velocities (*d* = 0.97, 0.78, and 0.29, respectively). Furthermore, the Hcon/Qecc ratio showed no difference (*p* > 0.05) between the performance groups in D (*d* = 0.05, 0.54, and 0.24, respectively) and ND (*d* = 0.69, 0.63, and 0.09, respectively) at three velocities.

## Discussion

The aim of the current study was to assess isokinetic knee strength profiles in boxers of different performance levels. The results confirmed our hypothesis. The RPT in hamstrings and quadriceps was stronger in the elite group than in the non-elite group. Elite boxers exhibited greater BLs than the non-elite group. Hcon/Qcon and Hecc/Qcon ratios may increase with angular velocity, regardless of the group. Hecc/Qecc and Hcon/Qecc ratios varied little with angular velocity.

### Peak torque

The relative peak torque of Hcon and Qcon was greater in the elite group in all angular velocities, and the value was higher in the D limb. The higher RPT in elite boxers might be due to optimal neuromuscular adaptations as a result of longer training experience (Quinzi et al., 2015). For boxers, the knee first initiates the action by applying force against the ground and then rotating the trunk and stretching the upper limb to punch (Lenetsky et al., 2013). In the present study, Qcon indicated a large effect size, which may be related to the kinematic and kinetic characteristics of the punch action. Electromyographic research suggested that activation of the rectus femoris extension (Qcon) and bicep femoris flexion is crucial for maximal force punching, especially the influence of rectus femoris (Qcon) activity on a powerful straight punch (Lenetsky et al., 2013). Boxers are required to have greater Qcon strength to promote greater ground reaction force. It has been reported that increasing ground reaction force generated by knee extension is conducive to improving punch force (Lenetsky et al., 2013). Similarly, it has been reported that Qcon strength contributes significantly to the throwing in the shot put (Łysoń-Uklańska et al., 2021). Also, Loturco et al. found a significant correlation in boxers between lower limb muscle strength and maximal punch force (Loturco et al., 2016). Therefore, to increase ground reaction force and improve punch force, heavy-load resistance training programs should be conducted, including traditional resistance exercises and complex training.

The change of RPT was not evident in the eccentric mode, regardless of the test speed. The RPT of Hecc and Qecc was greater in the elite than in the non-elite group. Interestingly, all boxers exhibited larger Qecc values in ND than D limb, except for at 240°/S. This might be because boxers need the powerful Q of ND limb to produce braking force, thus completing punching. The ground reaction force comes from Qecc contraction in the ND limb, which is similar to that in the javelin throw. Athletes transfer force to the upper limb by breaking the fall with a rigid ND limb. Briefly, the back-action force can transfer linear momentum into angular momentum, and the force will continue to transmitting by kinetic chain (Morriss et al., 2001). Javelin throwers land with a stronger front leg on the final stride, resulting in greater force being exerted on the javelin. For boxers, in order to produce more powerful punching, the greater strength of Qecc in the ND limb is required, which would result in a more effective blocking action. Athletes and coaches can use flywheel exercise to develop eccentric strength and optimize the benefits of resistance training.

### Bilateral ratios

Bilateral difference of oversizing is a known risk factor for muscle injury (Daneshjoo et al., 2013). BLs from 0 to 10% is considered within a normal range, and 10–20% is probably abnormal (Ellenbecker and Davies, 2000). BLs above 15% indicates leg strength imbalance and higher risk of knee-joint injury in the weakest limb (Croisier et al., 2008). In this study, some of the elite boxers (Qcon: 4, Qecc: 5) showed bilateral ratios greater than 15%. It is worth noting that BLs was greater in the elite than in non-elite group boxers because the BLs will increase as the training experience does (Lisowska et al., 2020). Boxing is a nonsymmetrical sport. The higher activity frequency and longer training durations of elite boxers may enhance their risk of injury. It has been noted that repeating the same movement patterns over a number of years might lead to negative consequences, which generally present as contralateral muscle strength imbalances (Probst et al., 2007; Tourny-Chollet et al., 2009).

Asymmetry is universal in boxers for two reasons. The first is the punching action caused by the way of force-generating. During the punch, there are distinct differences in muscle activation patterns between the D and ND limbs (Lenetsky et al., 2020). When the boxer performs punching, the knee in the D limb is mainly responsible for producing extension force. However, the supportive strength of the ND knee is to coordinate D knee motions, similar to the football player (Sangnier and Tourny-Chollet, 2007). The second reason may be the difference in frequency of using both legs. Compared to other sports, such as running and swimming, boxers have to perform offense or defense actions with varying movement frequencies in both legs. Prior studies have suggested restoring the weakest limb strength to within 10% of that of the unaffected limb (Kannus, 1994; Sherry and Best, 2004). Unilateral resistance training may contribute to reducing between-limb asymmetry (Gonzalo-Skok et al., 2017). Croisier et al. indicated that restoring a normal strength balance between agonist and antagonist muscles significantly decreases injury incidence (Croisier et al., 2008). Therefore, unilateral strength training should be supplemented with traditional bilateral exercise in order to improve maximal strength (Ramirez-Campillo et al., 2018) and reduce the risk of injury (Kovacs, 2006).

### Unilateral ratios

Hecc/Qecc ratios changed little in both groups. However, Hcon/Qcon tended to increase with the increasing velocity, which is consistent with the previous findings (Rosene et al., 2001; Holcomb et al., 2007). We found that this is due to significant decrease in strength produced by Qcon. However, the strength produced by Hcon did not vary significantly as the angular velocity increased. The Hcon/Qcon ratio increased substantially at higher velocity. Similarly, Hcon/Qcon ratios were smaller at higher velocity in elite boxers because they had greater ability to maintain the strength of Qcon compared with the non-elite group. The reasonable explanation for this is that hamstring muscles have faster muscle fibers relative to quadriceps muscles (Garrett et al., 1984).

Unilateral ratios are an important predictor of injury (Kong and Burns, 2010; Orchard et al., 2012). Maintenance of the balance between H and Q strength is conducive to the stability of knee joints. It was reported that the normal value of Hcon/Qcon is higher than 0.6, and if it is lower than 0.6, the imbalance between the flexion and extension will lower the stability and increase the risk of injury 17 times (Coombs and Garbutt, 2002; Zvijac et al., 2014). In the studies examined by Kannus (Kannus, 1988a), healthy H: Q ratio recommendations varied from 0.50 to 0.80. Orchard et al. (1997) suggested a conventional ratio at least 0.61 and Dunnam et al. (1988) proposed 0.6 to prevent ACL injuries. In this study, Hcon/Qcon ratios were less than 0.6 at 60°/S, but higher at the other two velocities. In addition, Hcon/Qcon and Hecc/Qecc ratios were lower in the elite group than in the non-elite group at all velocities, suggesting that elite boxers require hamstring-specific resistance training to match the knee extension moments (Holcomb et al., 2007).

### Functional ratios

It is well known that co-activation of the flexors and extensors occurs by opposing contraction patterns. Thus, the functional ratio could help accurately assess muscle function (Coombs and Garbutt, 2002). The Hecc/Qcon ratio has a stabilizing effect during movement of knee extension (Opar and Serpell, 2014), and the ideal range is 0.8–1.2 (Delextrat et al., 2010). A previous study showed that a healthy Hecc/Qcon functional ratio is 1.0 or 1:1 (Aagaard et al., 1998). Li et al. also indicated that a Hecc/Qcon of 1.00 is advantageous for limiting anterior tibial translation in knees with ACL deficiency (Li et al., 1996). In the present study, Hecc/Qcon ratios were less than 1.0 at lower velocity in both groups. The higher the angular velocity, the larger the Hecc/Qcon values for boxers. We found a great decline in Qcon, but the value of Hecc did remain relatively constant as the velocity increased. This may be because the elastic component of muscle makes a major contribution to ecc contraction, which does not occur during ecc contraction. With increasing velocities, Colliander et al. discovered that Qcon and Qecc peak strength decreased at a faster rate than Hcon peak strength. This faster decline of Qcon strength in comparison to Hecc led to a increase in Hecc/Qcon ratios (Colliander and Tesch, 1989). Furthermore, the Hecc/Qcon ratio was significantly lower (*p* < 0.01) in the elite group than in the non-elite group at 180D and 240D. Elite boxers require the production of stronger Qcon to enhance ground reaction force to improve punch force (Lenetsky et al., 2020), which also sets a higher requirement for the Hecc. To reduce the risk of H strain, it is necessary to increase the Hecc strength of elite boxers to better match higher Qcon. A Hcon/Qecc ratio represents the function of knee flexion motion. The ratios for boxers were 0.36–0.57, which were in accordance with the normal range (0.28–0.60) in a previous study. This is because the contractility of Hcon is weaker with respect to Qecc (Coombs and Garbutt, 2002).

Both Hcon/Qcon and Hecc/Qcon ratios were higher in the ND limb than in the D limb. The nature of boxing provides an explanation for the ND limb generating higher ratios, in that the ND limb functions as the stabilizing limb more often. During punch movement, the boxer moves toward the target with the vertical force generated by the D limb extension to move their body forward. Subsequently, the ND limb (lead leg) makes contact with the ground, bearing body weight and maintaining stable body posture (Lenetsky et al., 2020). This necessitates dynamic regulation of lower limb deceleration by hip and knee muscles. Mainly, the hamstrings and quadriceps function together to maintain this load-bearing capability (Griffin et al., 2000). To improve punch force, maximal knee extension and hip flexion while performing the punch are necessary in the D limb (Turner et al., 2011). These movements are likely to be repeated hundreds of times in one training session. This frequent Q action may result in muscle strength asymmetries. Concordantly, Hcon/Qcon and Hecc/Qcon ratios were lower in the D limb than in the ND limb.

The current study had some limitations. First, the sample size was small because few athletes from a single region met the rigorous inclusion criteria in the study. Second, five contractions were performed in familiarization sessions, not making 5–10 submaximal repetitions, which is a method based on findings of a previous study (Chan et al., 2020). According to the method described by Chan et al., no earlier submaximal contractions were performed because this could have caused confusion with respect to the number of sets required for familiarization, and the attribution of any changes solely to maximum contractions.

## Conclusion

Relative peak hamstring and quadricep torque during con and ecc contractions was higher in the elite group than in the non-elite group. Boxers had a normal Hcon/Qecc ratio at the UL motion. Hcon/Qcon and Hecc/Qcon ratios were below standard values at lower angular velocity, suggesting improved Hcon and Hecc strength. Elite boxers had greater BLs, indicating that bilateral strength differences may increase with training experience. Overall, elite boxers had lower ULs and functional ratios, indicating a higher risk of injury. In order to prevent injury and induce better performance, it is necessary to improve hamstring strength and periodically evaluate asymmetries of strength in boxers. The results of this study provide empirical support for lower-limb strength training in boxers.

## Data Availability

The original contributions presented in the study are included in the article/Supplementary Material; further inquiries can be directed to the corresponding author.
